# Analysis of the Physical and Structure Characteristics of Reformulated Pizza Bread

**DOI:** 10.3390/foods11131979

**Published:** 2022-07-04

**Authors:** Syed Saif Alam, Deepti Bharti, Bikash K. Pradhan, Deblu Sahu, Somali Dhal, Nahyun Mariah Kim, Maciej Jarzębski, Kunal Pal

**Affiliations:** 1 Department of Biotechnology and Medical Engineering, National Institute of Technology Rourkela, Rourkela 769008, Odisha, India; saiftx@gmail.com (S.S.A.); deeptibharti94@gmail.com (D.B.); bikashpradhan21@gmail.com (B.K.P.); deblusahu.nit@gmail.com (D.S.); somali.dhal12@gmail.com (S.D.); 2 Riverside School of Medicine, University of California, 900 University Ave, Riverside, CA 92521, USA; nkim133@medsch.ucr.edu; 3 Department of Physics and Biophysics, Faculty of Food Science and Nutrition, Poznań University of Life Sciences, Wojska Polskiego 38/42, 60-637 Poznań, Poland

**Keywords:** pizza base, white flour, whole wheat flour, dietary fiber, texture

## Abstract

The current study deciphers the processing of different proportions of white flour and whole wheat flour (100:0, 75:25, 50:50: 25:75, and 0:100) into a pizza base using yeast-based fermentation. The bread making using the yeast system resulted in significant changes in the characteristics of bread, ranging from the porous structure development to the crumb cellular structure modifications. An increase in the proportions of whole wheat flour resulted in the formation of golden yellow pizza bases. The lightness of the crust was decreased, whereas the yellowness index was increased as the whole wheat flour contents were increased. The pore size of the pizza base was decreased while the pore density was increased as the whole wheat flour content was raised within the bread. The microscopic study also showed the formation of porous structures on the bulk of the pizza base. The texture analysis of the bread also suggested an increase in the formation of the rigid network structure when the amount of whole wheat flour was increased. The springiness, cohesiveness, and resilience were comparable for all the prepared samples. On the other hand, the values for hardness, gumminess, and chewiness showed an increasing trend with the increase in the whole wheat flour content. The impedance of the samples decreased when there was an increase in the whole wheat flour content. Overall, the pizza base that was developed with 50% whole wheat flour and 50% white flour ratio displayed acceptably firm yet sufficient viscoelastic properties for human consumption.

## 1. Introduction

The modern pizza, as we know it today, originated in Naples, Italy, as an affordable, fast meal for working-class Neapolitans; however, the pizza in its most basic form as a seasoned flatbread has now been popularized worldwide, with different cultural versions of pizza based on local ingredients and flavors [1]. A continuous rise in pizza consumption led to an increase in the number of consumers and, thereby, pizza outlets in many countries. This increase corresponds to more than 1.5 times in 12 years [2]. Hence, statistically, it can be safely deduced that pizza consumption has increased considerably over the last decade and that the pizza dough has evolved into one of the world’s most beloved baked foods [3]. Pizza is considered a one-stop meal for the entire dietary requirement whose ingredients include carbohydrates, proteins, and vitamins in the diet [4]. The pizza base is a flatbread that can be leavened by chemical processes or yeast. Flatbread is made from milled cereal grains, with wheat being one of the most commonly used grains. The unique interaction between the proteins in milled wheat flour and water makes this grain a versatile ingredient for bread-making [5].

The pizza base is made predominantly from refined wheat grain flour, which is also known as all-purpose flour or white flour (WF) [6]. The wheat grain consists of layers that can be broadly classified as the outer bran, endosperm, and germ layer [7]. The pizza base is made predominantly from refined wheat grain flour. Although wheat grain has many beneficial properties as a staple consumable, a substantial amount is lost with the removal of the bran layer [8]. The WF is composed of a starch core with a surrounding protein matrix at the microscopic level. This matrix consists mainly of gliadin and glutenin, also known as gluten, which is insoluble in water [9]. It has been seen that a larger concentration of gluten is linked to an appreciable decrease in the absorption of carbohydrates in the digestive system, resulting in flatulence and bowel discomfort in the human body [10].

This phenomenon may be avoided by using WF in conjunction with whole wheat flour (WWF) [10]. While the carbohydrates in the wheat flour are an abundant source of energy, the bran layer of the wheat grain is rich in dietary fibers and contains various minerals that express antioxidant activity (e.g., potassium (K), calcium (Ca), manganese (Mn), phosphorous (P), magnesium (Mg), and sodium (Na)) and phytochemicals (e.g., phenolic acid, carotenoid, and tocopherol [11]. The minerals present in the bran also help in preventing chronic illnesses resulting from the deficiency of macro and micro [12].

Many studies have attempted to analyze the changes in the structural and physical properties of food products due to WWF substitution. The alteration in the antioxidant (e.g., carotene) and carotenoid (e.g., lutein) concentrations due to the inclusion of the bran fraction of whole wheat into white flour has been studied [13]. Another study aimed to enhance the nutritional benefits of steamed bread usually made with WF by including bran. Similarly, researchers have also studied the changes in the dough rheology and the technological properties of bread by including different bran fractions [14]. Accordingly, in the present research, we studied the changes in the physical characteristics of the pizza base by substituting WF with WWF in differing proportions [15]. The prepared pizza base was initially characterized based on the color parameters through the colorimetry device. Further, the base was evaluated for the pore size and distribution within its bulk phase, which may assist in its texture. The textural properties of the pizza bases were characterized by stress relaxation and texture profile analyses. The observed pore size distribution was also correlated with the impedance spectroscopy data study.

## 2. Materials and Methods

### 2.1. Materials

Whole grain wheat flour (Ashirvaad Shudh Chakki Atta, Swastik Grain Pvt. Ltd., Bangalore, India), all-purpose flour (generic brand), baking powder (Weikfield foods Pvt. Ltd., Pune, India), salt (TATA Vacuum evaporated Iodized Salt, Tata chemical Ltd., Dwarka, India), sunflower oil (Fortune, Adani Wilmar Pvt. Ltd., Mangalore, India), and active dry yeast (generic brand) were sourced from the local market.

### 2.2. Sample Preparation

The pizza base was prepared by mixing and kneading the wet and dry ingredients to form a dough. The dough was developed using different WF and WWF proportions. To the mixture, salt and baking powder were added. The ingredients were then mixed thoroughly for a minute with a whisk to ensure the formation of a homogenous mixture. Subsequently, water was warmed to bloom the yeast (37–45 °C). Accurately weighed active dry yeast and coarse grain sugar were added to the lukewarm water. The mix was then set to rest for 5 min. The bloomed yeast obtained was used for kneading dough. The process of kneading continued for 15 min. The finished dough was then kept in an oiled container, covered with cling wrap, and incubated for 3 h at room temperature (25 °C). After the incubation period, the dough was stretched out in a circular shape on a baking pan. The baking pan was then kept in the oven (Samsung Smart Oven–MC32J7035CT, Samsung Electronics Pvt. Ltd., Kuala Lumpur, Malaysia), which was pre-heated in the convection mode at 200 °C. Thereafter, baking was carried out at the said temperature for 12 min. At the end of the baking period, the finished pizza base was taken out and allowed to cool for 5 min. The composition of the prepared pizza bases is tabulated in Table 1.

### 2.3. Mehods

#### 2.3.1. Color Analysis

The colorimetric study was performed on the pizza base crust formulations using a lab-developed colorimeter to acquire the CIELab (L*, a*, and b*) values. The detailed principle and working of the colorimeter are reported in the mentioned study [16]. Initially, the calibration of the colorimeter was performed with black and white tiles. The samples (cylindrical shaped; Diameter: 35 mm, Height: 10 mm) were taken in a 35 mm petri dish, and the surface images were acquired by the colorimeter L* (lightness), a* (values ranging from red to green) b* (values ranging from yellow to blue) were then calculated by the colorimeter. The whiteness (WI) and yellowness (YI) indices of the samples were calculated from the acquired L*, a*, and b* data using the given formulae [17]:

(1) $$\mathrm{WI}=100-\sqrt{{\left(100-L^{*}\right)}^{2}+{\left(a^{*}\right)}^{2}+{\left(b^{*}\right)}^{2}}$$

(2) $$\mathrm{YI}=142.86\times \frac{b^{*}}{L^{*}}$$

#### 2.3.2. Microscopic Analysis

The surface topology of the crust and the bulk of the rectangular pizza base samples (length and width: 15 mm) were visualized using a Stereo Zoom Microscope (Model: SM-2TZ; Make: AMscope, Irvine, CA, USA). The microscope used an external eyepiece lens camera (AMscope MD500, Irvine, CA, USA) to acquire images.

### 2.4. Texture Analysis

The samples were subject to texture analysis to characterize the viscoelastic and textural properties.

#### 2.4.1. Stress Relaxation

Stress relaxation of the samples was performed to characterize the viscoelastic properties. The experiment was carried out using a texture analyzer (model: Texture analyzer HD plus; Stable Micro Systems, Godalming, UK). The pizza bases were cut into individual rectangular cuboid pieces (length: 15 mm, width: 15 mm, and height: ~20 mm). The stress relaxation was performed with the flat probe (diameter: 63.5 mm). The probe pressed the samples down by a distance of 2 mm at a speed of 1 mm/s after a trigger force of 5 g. The deformation was then maintained for the 60 s, and the relaxation profiles were obtained by monitoring the force values over time. The probe was then redacted to its original height.

#### 2.4.2. Texture Profile Analysis

Texture profile analysis was performed to study the various textural parameters such as hardness, springiness, cohesiveness, gumminess, chewiness, and resilience. The flat probe (diameter: 2.5 inch) was used to apply 50% strain to the sample for two compression cycles at a speed of 1 mm/sec. A time gap of 5 sec was maintained between the two cycles.

#### 2.4.3. Impedance Analysis

Impedance analysis of the pizza base samples was performed using an impedance analyzer (Diligent, Analog Discovery 2, National Instruments, Austin, USA). The sample was kept on an insulator (cardboard), and the impedance probe (circular shaped; Diameter: 10 mm, Distance between electrodes: 10 mm) was inserted into the bulk of the samples. The impedance was measured at the frequency range of 1 Hz to 1 KHz.

#### 2.4.4. Date Representation

Data of different studies are represented as mean ± S.D of the triplicates.

## 3. Results and Discussion

### 3.1. Visual and Physical Appearance

**Figure 1:**
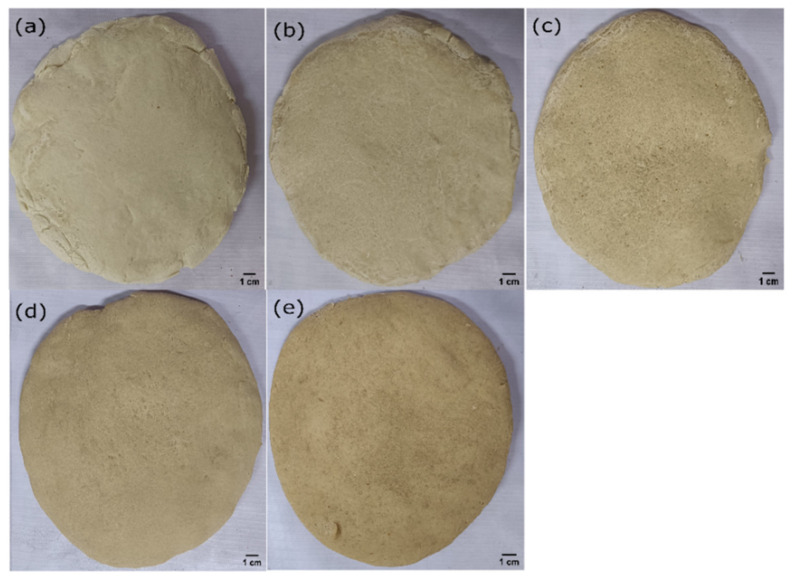
The top view of different samples. (**a**) WF0, (**b**) WF25, (**c**) WF50, (**d**) WF75, and (**e**) WF100 (Scale bar 1 cm).

**Figure 2:**
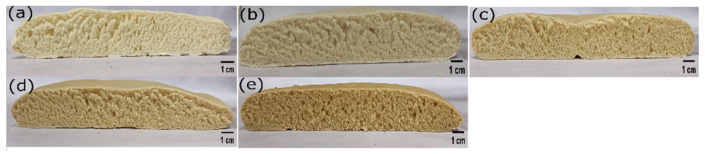
The cross view of different samples. (**a**) WF0, (**b**) WF25, (**c**) WF50, (**d**) WF75, and (**e**) WF100 (Scale bar 1 cm).

The differently formulated pizza bases were set to rest for 5 min after the baking process was complete. This was performed to ensure that the pizza bases reached a temperature that was safe to handle. The crust and bulk phase of the pizza bases were then imaged (Figure 1 and Figure 2), with the sample being kept 300 mm away from the pizza base formulation. Visually, the formulation WF0, which had no WWF, appeared whitish. With a progressive replacement of WF with WWF (i.e., WF25, WF50, WF75, and WF100), the subsequent formulations started appearing golden yellow. This change of color was attributed to the presence of lutein, as illustrated by various studies [13,14]. Moreover, the crust was smooth to touch, and the perception of touch was similar in all the samples. The visual impression of the bulk phase suggested that the pore size was decreased, with a consequent increase in the pore density as the replacement proportion of WWF was increased. A study suggested that the interaction between the whole wheat fiber and the gluten matrix results in a decrease in the ability of the dough to retain gas. This phenomenon results in the formation of denser and smaller pores in the dough [14].

### 3.2. Colorimetry

**Figure 3:**
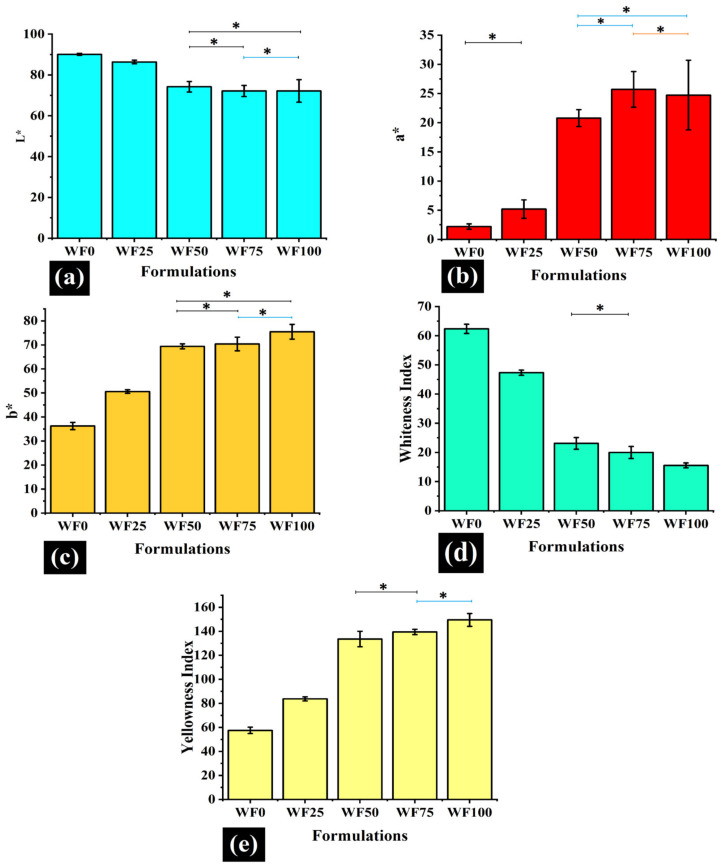
The colorimetric analysis is illustrated in bar graphs. (**a**) L* values of the different formulations. (**b**) a* values of the formulations. (**c)** b* values of the formulations. (**d**) Whiteness index of samples. (**e**) Yellowness index of the samples. (Formulations with * sign have similar values; *p* > 0.05).

Color analysis of a food product is an essential step that accounts for a crucial sensory characteristic. The CIELab color-space or L*a*b* color-space was developed by Commission Internationale de l’Eclairag, which defined and standardized a color measurement protocol that has been used for the color analysis of food products [18]. The CIELab color space extends beyond RGB (red, green, and blue) and CMYK (cyan, magenta, yellow, and black) color space in the sense that it also includes a lightness component. L* (lightness or luminous) values are gray values that range from 0 (black) to 100 (white). a* and b* are the chromatic components in the CIELab color space and range from −120 to +120. Moving along the positive a* axis represents a color shift to red, and the negative a* axis represents a color change to green. Similarly, moving in the positive b* axis represents a shift to yellow, and moving in the negative b* axis represents a change to blues [17,19]. The average values for the L * components for all formulations were found between 72 and 91 (Figure 3a), which suggests that the prepared samples were quite luminous. The L* values of the samples decreased with an increase in WWF proportion until WF50. A further increase in WWF did not result in significant changes in the L* values as compared to WF50 (Figure 3a). The negative correlation of L* with the increasing whole wheat fraction is in accordance with the previous study [13], wherein the authors analyzed the effect of the addition of different proportions of bran fractions in WF. This negative correlation of L* with a progressively increasing percentage of bran fraction has been reported previously [14].

The average a* values of the samples were in the range of 2 and 25 (Figure 3b). This is suggestive of the presence of a red shade in the samples. An increase in the WWF proportion progressively increased the a* values. This suggests that the addition of WWF increased the red component within the samples. This positive correlation of a* values and WWF proportion is attributed to the increase in lutein concentration in the samples with a corresponding increase in the WWF proportion [13]. The average b* values (Figure 3c) of the samples were in the range of 36 and 76, an indication of a yellow hue. The b* values showed a considerable increase in b* values as we progressed from the lowest concentration of WWF to that of the highest. The lutein content imparts the yellow color to wheat, which can also explain this observation [13]. In [13], it has been reported that there is a positive relationship between the lutein content and the yellow color.

The above values (L*, a*, and b*) were used to calculate derived parameters such as whiteness index (WI) (Figure 3d) and yellowness index (YI) (Figure 3e). The WI values ranged from 15 to 63, displaying an overall decreasing trend with an increased proportion of WWF. On the contrary, YI values ranged from 57 to 150, which displayed a consistent increase in the yellowness of the samples as WWF proportion was increased. The presence of lutein, which imparts a yellow color, can be linked to the increase in YI [13]. Overall, it is quite evident that the changes in the color attributes of the samples can be associated with the lutein content, a major carotenoid present in whole wheat [13].

### 3.3. Microscopic Studies

**Figure 4:**
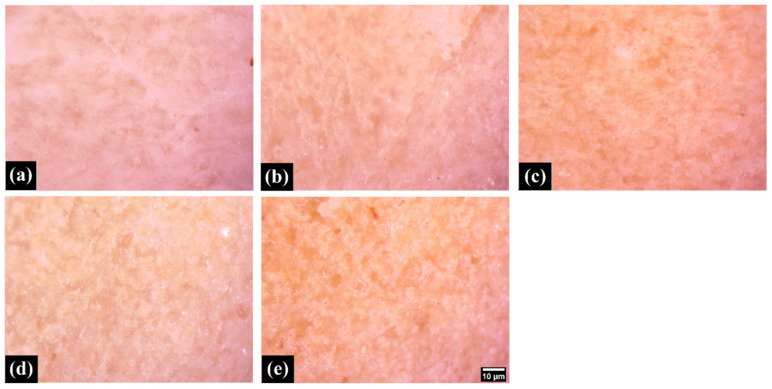
The micrographs of the various samples show the samples’ crust. (**a**)WF0; (**b**) WF25; (**c**) WF50; (**d**) WF75; (**e**) WF100. (Scale bar: 10µm—the same magnification for all images).

**Figure 5:**
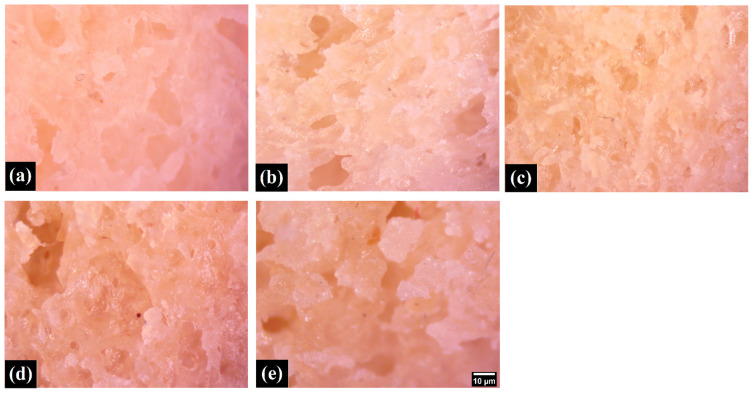
The micrographs of the various samples show the cross-section of the samples. (**a**) WF0; (**b**) WF25; (**c**) WF50; (**d**) WF75; (**e**) WF100. (Scale bar: 10µm—the same magnification for all images).

Microscopic studies were carried out to visualize the surface microtopography of the crust and the bulk of the samples. The crust (Figure 4) showed the presence of corrugations, which increased from WF0 to WF100. The presence of the bran component can explain this increase in the corrugations as WWF is increased. The cross-section (Figure 5) of samples (bulk phase) was also visualized under the stereomicroscope. The topography of the samples suggested the formation of an overall porous matrix. The overall pore size and density at the microscopic level appeared similar in all the samples. Data are represented as mean ± SD of triplicates (* represents *p* > 0.05).

In [20], the authors applied scanning electron microscopy (SEM) for the freeze-dried samples of pizza crust baked in ovens. SEM analysis has also been proposed for the evaluation of cooking quality [21]. Nevertheless, performed light microscopy is beneficial due to the possibility of evaluating larger parts of pizza bread. Further studies might be interesting for detailed correlation between microscopic analysis, as performed here, with texture. Here, we would like to highlight that the light microscopy results corresponded with the results performed by electrical impedance spectroscopy. The porosity of the pizza bread correspondingly affected their impedances (for details, see p. 4.6)

### 3.4. Stress Relaxation (SR)

**Figure 6:**
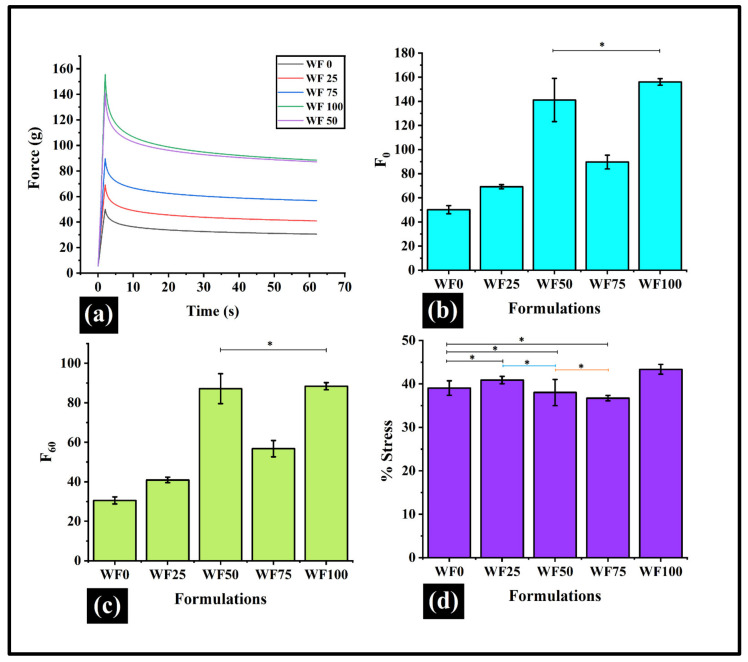
Graphs representing stress relaxation parameters: (**a**) The stress relaxation (**b**) F0; (**c**) F60; (**d**) %SR. The curve is illustrated as a line graph. Data are represented as mean ± SD of triplicates (Formulations with * sign have similar values; *p* > 0.05).

SR is performed for many food products to characterize their viscoelastic properties. The highest force attained in the SR profile is used to forecast the firmness of the formulations (F0). The F0 values (Figure 6b) showed an increasing trend from WF0 to WF100. Interestingly, WF50 has shown an abrupt rise in the F0 value from the rest of the samples, except WF100. The F0 value of WF50 and WF100 were similarly (*p* > 0.05). The unusual F0 value of WF50 can be explained by the formation of some associative interactions between gluten and dietary fiber. Further, the increased firmness of the WWF-containing pizza base can be explained by the increase in the bran fraction [22]. The bran contains dietary fiber, which increases dough structure and quality, but excess amounts of this dietary fiber interfere with the gluten network formation, thereby making the dough denser [20]. As the strained condition was maintained for 60 sec, the force values exponentially dropped with time after reaching the F0. The force value at the end of the relaxation profile was marked as F60. The F60 values (Figure 6c) showed a similar trend to F0. F60 of WF50 also showed an unusually high value compared to other samples, confirming its criticality.

The F0 and F60 were further used to calculate the %SR for all the formulations. The %SR values (Figure 6d) of WF0, WF25, WF50, and WF75 were comparable. This showed that the stress values developed in these samples were somewhat similar. A similar %SR value of WF50 and WF0, even though the F0 value of WF50 was quite high. This can be explained by the increase in the rigidity of the WF50. As WF50 was put under strain, the rigid structure started to break down inhomogeneously. Due to this inhomogeneous breaking of the network structure, an increased standard deviation was observed for the F0 and F60 values. On the contrary, WF100 showed a substantial rise in the %SR from WF0. A higher %SR value of WF100 and a lower standard deviation for the F0 and F60 values are indicative of a rigid system that underwent quick and homogenous network breakage.

### 3.5. Texture Profile

**Figure 7:**
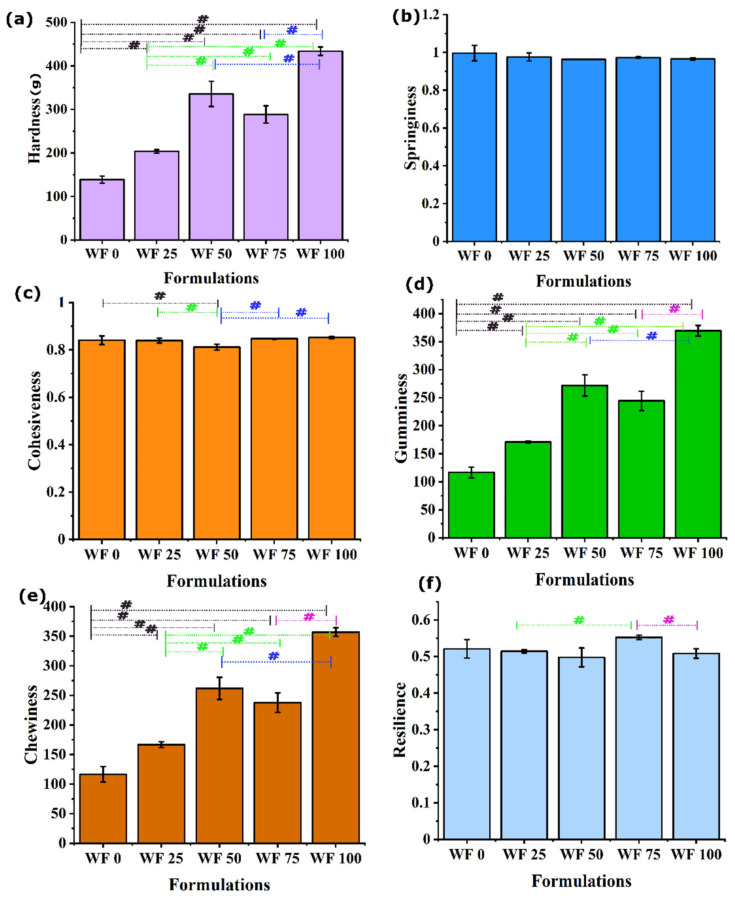
Graphs representing parameters obtained from TPA: (**a**) Hardness; (**b**) springiness; (**c**) cohesiveness; (**d**) gumminess; (**e**) chewiness; (**f**) resilience. Data are represented as mean ± SD of triplicates (# *p* < 0.05).

Texture profile analysis was performed on the pizza base crumb to extract six crucial parameters, i.e., hardness (g), springiness, cohesiveness, gumminess, chewiness, and resilience. Hardness refers to the force required to achieve a particular deformation [23]. The hardness of all the formulations ranged from 130 g to 440 g (Figure 7a). There was an overall increase in the hardness with a corresponding increase in WWF content. The positive correlation of hardness with an increasing percentage of whole wheat can be attributed to an increased concentration of dietary fibers [22]. This is due to the fact that the added bran part introduced dietary fiber that interacted with the gluten network, eventually making the network denser [22]. In addition, the bran might have behaved as a reinforcing agent, which may have played an important role in improving the hardness. Springiness is yet another aspect of textural quantification that aids in maintaining the quality and shelf life of food products [24]. It is the measure of the rate at which the deformed material comes back to the undeformed shape. These values (Figure 7b) did not differ much among the samples, suggesting that replacing WF with WWF had little impact on the springiness of the pizza base.

Another parameter that was calculated from the TPA analysis was cohesiveness, the degree to which a material is fully compressed between teeth before rupturing. The cohesiveness values ranged from 0.80 to 0.86, as illustrated in (Figure 7c). It can be noted that the values of cohesiveness for all the formulations are comparable and did not change appreciably. The property of gumminess is defined as the energy necessary to break down a semi-solid material into a swallowable state; however, chewiness is defined as the amount of time it takes to chew a sample at a constant rate of force until it reaches the right consistency for swallowing. The values of gumminess (Figure 7d) and chewiness (Figure 7e) increased monotonously from WF0 to WF100, with an abrupt higher value for WF50. The parameters of the gumminess and chewiness played an essential role for different age groups of consumers.

The last parameter of TPA was resilience, defined as the degree of resistance a material gives against deformation [25,26]. The values for resilience (Figure 7f) were in the range of 0.52 and 0.56. Resilience for all of the samples was comparable. A similar result was reported in [14], where bread was reformulated using bran fractions.

### 3.6. Impedance Analysis

**Figure 8:**
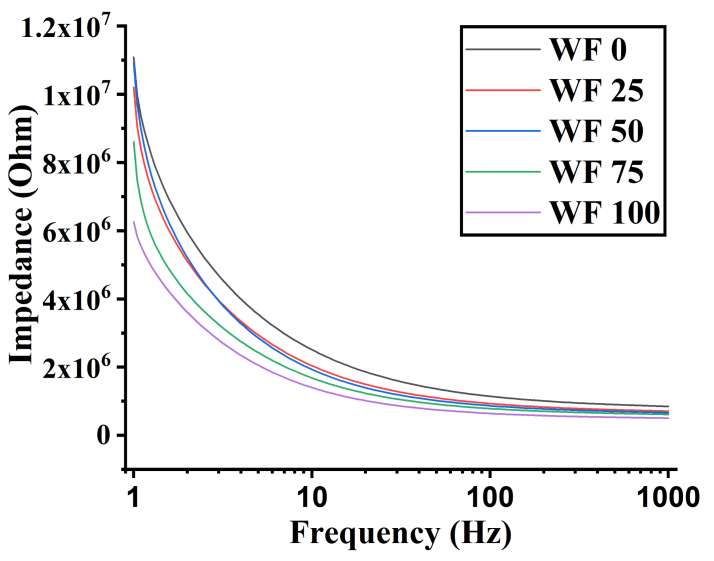
Graphs representing impedance of various samples.

Electrical impedance spectroscopy (EIS) is a technique that is used to measure the electrical characteristics of materials by applying a sinusoidal test voltage or current across the bulk. The impedance plots (Figure 8) show a decrease in impedance values with the subsequent replacement of WF with WWF. Because the pizza crumb is a porous material, as confirmed by microscopic analysis, the decrease in impedance is thought to be based on factors such as pore size, pore density, and water content [27]. As mentioned in Section 3.1, there was an increase in the pore density with a corresponding decrease in the pore size as the WWF content was increased. This can account for the decrease in the impedance values as the WWF content was increased. A correlation between electrical impedance spectroscopy with pore size and density has been analyzed [28]. The increased mineral (Na, Mn, K, Ca, Zn, Fe, and Cu) content can also be associated with the decreased impedance as the WWF content was increased. The mineral and dietary fiber have a water holding capacity and can lead to the formation of ions upon coming in contact with water which increases the sample’s conductivity and thus reduces impedance [29].

Furthermore, it should be noted that bakery products and the pizza bread manufacturing industry are automated, including high-speed production lines [30]. The quality control of the bakery products is crucial for their safety and consumers’ satisfaction. From that point of view, EIS might be a new tool for the quality check of pizza bread and other baking products.

## 4. Conclusions

The continuously changing human lifestyle has also changed the nature and globalization of food by demanding healthier qualities and nutritional values. This has also been portrayed in the worldwide tradition of flatbreads to the modern pizza as we know it today, due to its ease in preparation, portability, affordability, easy customization, and balanced composition. In most cases, the basic structure of pizza is based upon the bread prepared using WF; however, unbeknownst to many, the gluten content in WF brings both benefits and harm to the human body. For instance, the long-term and repetitive consumption of gluten is associated with allergic reactions, irritable bowel syndrome, gluten ataxia, etc. A gluten-free diet is the most widely recommended, and a large number of gluten-free products (GFP) are available on the market. These GFPs arguably mimic the macronutrient and dietary fiber composition of their gluten-containing counterparts (GCC) [31]. A cross-sectional study analyzing the nutritional differences between hundreds of GFP and GCC demonstrated that the latter had higher protein content, especially in flour, bread, pasta, and pizza [31,32]; therefore, replacing WF with WWF is a straightforward method to improve the nutritional properties of pizza bases. The current study replaced WF with WWF (100:0, 75:25, 50:50: 25:75, and 0:100) in the pizza base. The base was formulated using yeast-based fermentation and was further studied to obtain substantial changes in the characteristics of bread. It was visually evident that the replacement of WF with WWF resulted in the development of golden yellow pizza bases. The color studies confirmed a decrease in the lightness of the crust and increased yellowness index upon continuous replacement of WF with WWF. It was observed that the pore size of the pizza base was decreased while the pore density was increased as the WWF content was increased within the bread. The microscopic study further confirmed the porous structure of the crumb of the pizza base. The structure porosity had the potential to affect the texture. The texture studies of the bread suggested increased structural rigidity when the amount of WWF was increased. The springiness, cohesiveness, and resilience were comparable for all the prepared samples. Interestingly, the values for hardness, gumminess, and chewiness showed an increasing trend with the increase in the WWF content. These texture properties were crucial and yet essential to fulfill the requirements of a large age group population. The increased content of WWF resulted in decreased impedance. Altogether, the pizza base that was developed using 50% of WWF and 50% of WF exhibited reasonably firm yet sufficient viscoelastic properties. These results are quite convincing that modifications to the (WF and WWF ratio in pizza bread dough) may be recommended to navigate the world towards a new era of healthier nutrition and delicious pizza-making. Finally, we opened a way for designing a new tool for food quality evaluation based on EIS possible application for baking products.

## Figures and Tables

**Table 1:** Compositions of the pizza base.

Formulations	Composition (g)						
	WF	WWF	Salt	Baking Powder	Yeast	Water	Oil
WF0 (Control)	240	0	1.6	0.18	1	125	4.3
WF25	180	60	1.6	0.18	1	125	4.3
WF50	120	120	1.6	0.18	1	125	4.3
WF75	60	180	1.6	0.18	1	125	4.3
WF100	0	240	1.6	0.18	1	125	4.3

## Data Availability

The data presented in this study are available on request from the corresponding author.

## References

[1] Helstosky C. (2008). Pizza: A Global History.

[2] Statista Domino’s Pizza Stores in Selected Countries 2020. https://www.statista.com/statistics/207130/number-of-dominos-pizza-stores-worldwide-by-country/.

[3] Moore J., Luther M., Cheng Z., Yu L. (2009). Effects of baking conditions, dough fermentation, and bran particle size on antioxidant properties of whole-wheat pizza crusts. J. Agric. Food Chem..

[4] Redl A., Guilbert S., Morel M.-H. (2003). Heat and shear mediated polymerisation of plasticized wheat gluten protein upon mixing. J. Cereal Sci..

[5] Catterall P., Cauvain S.P. (2007). Flour Milling. Technology of Bread-Making.

[6] Tehseen S., Anjum F.M., Pasha I., Khan M.I., Saeed F. (2014). Suitability of spring wheat varieties for the production of best quality pizza. J. Food Sci. Technol..

[7] Zhu Y., Sang S. (2017). Phytochemicals in whole grain wheat and their health-promoting effects. Mol. Nutr. Food Res..

[8] Pourafshar S., Rosentrater K.A., Krishnan P.G. (2015). Using alternative flours as partial replacement of barbari bread formulation (traditional Iranian bread). J. Food Sci. Technol..

[9] Biesiekierski J.R. (2017). What is gluten?. J. Gastroenterol. Hepatol..

[10] Anderson I.H., Levine A.S., Levitt M.D. (1981). Incomplete absorption of the carbohydrate in all-purpose wheat flour. New Engl. J. Med..

[11] Almeida E.L., Chang Y.K., Steel C.J. (2013). Dietary fibre sources in bread: Influence on technological quality. LWT Food Sci. Technol..

[12] Choi I., Kang C.-S., Hyun J.-N., Lee C., Park H.-G. (2013). Mineral compositions of Korean wheat cultivars. Prev. Nutr. Food Sci..

[13] Humphries J.M., Graham R.D., Mares D.J. (2004). Application of reflectance colour measurement to the estimation of carotene and lutein content in wheat and triticale. J. Cereal Sci..

[14] Blandino M., Sovrani V., Marinaccio F., Reyneri A., Rolle L., Giacosa S., Locatelli M., Bordiga M., Travaglia F., Coïsson J.D. (2013). Nutritional and technological quality of bread enriched with an intermediated pearled wheat fraction. Food Chem..

[15] Tritt A., Reicks M., Marquart L. (2015). Reformulation of pizza crust in restaurants may increase whole-grain intake among children. Public Health Nutr..

[16] Jain A., Pradhan B.K., Mahapatra P., Ray S.S., Chakravarty S., Pal K. (2021). Development of a low-cost food color monitoring system. Color Res. Appl..

[17] Sahu D., Bharti D., Kim D., Sarkar P., Pal K. (2021). Variations in Microstructural and Physicochemical Properties of Candelilla Wax/Rice Bran Oil–Derived Oleogels Using Sunflower Lecithin and Soya Lecithin. Gels.

[18] Leon K., Mery D., Pedreschi F., Leon J. (2006). Color measurement in L* a* b* units from RGB digital images. Food Res. Int..

[19] Youssef M., Barbut S.S. (2011). Fat reduction in comminuted meat products-effects of beef fat, regular and pre-emulsified canola oil. Meat Sci..

[20] Mastrascusa D., Vázquez-Villegas P., Huertas J.I., Pérez-Carrillo E., Nevarez R. (2022). Determination of pizzas quality and acceptability by physic-mechanical tests. J. Food Sci. Technol..

[21] Blutinger J.D., Meijers Y., Chen P.Y., Zheng C., Grinspun E., Lipson H. (2018). Characterization of dough baked via blue laser. J. Food Eng..

[22] Wu M.-Y., Chang Y.-H., Shiau S.-Y., Chen C.-C. (2012). Heology of fiber-enriched steamed bread: Stress relaxation and texture profile analysis. J. Food Drug Anal..

[23] Peleg M. (2019). The instrumental texture profile analysis revisited. J. Texture Stud..

[24] Pehlivanoglu H., Ozulku G., Yildirim R.M., Demirci M., Toker O.S., Sagdic O. (2018). Investigating the usage of unsaturated fatty acid-rich and low-calorie oleogels as a shortening mimetics in cake. J. Food Processing Preserv..

[25] Szczesniak A.S. (2002). Texture is a sensory property. Food Qual. Prefer..

[26] Yildiz Ö., Yurt B., Baştürk A., Toker Ö.S., Yilmaz M.T., Karaman S., Dağlıoğlu O. (2013). Pasting properties, texture profile and stress–relaxation behavior of wheat starch/dietary fiber systems. Food Res. Int..

[27] Kertész Á., Hlavacova Z., Vozáry E., Staronova L. (2015). Relationship between moisture content and electrical impedance of carrot slices during drying. Int. Agrophysics.

[28] Cabeza M., Keddam M., Nóvoa X.R., Sánchez I., Takenouti H. (2006). Impedance spectroscopy to characterize the pore structure during the hardening process of Portland cement paste. Electrochim. Acta.

[29] Alshehry G.A. (2020). Preparation and nutritional properties of cookies from the partial replacement of wheat flour using pumpkin seeds powder. World.

[30] Liberopoulos G., Tsarouhas P. (2005). Reliability analysis of an automated pizza production line. J. Food Eng..

[31] Calvo-Lerma J., Crespo-Escobar P., Martínez-Barona S., Fornés-Ferrer V., Donat E., Ribes-Koninckx C. (2019). Differences in the macronutrient and dietary fibre profile of gluten-free products as compared to their gluten-containing counterparts. Eur. J. Clin. Nutr..

[32] Cornicelli M., Saba M., Machello N., Silano M., Neuhold S.J.D., Disease L. (2018). Nutritional composition of gluten-free food versus regular food sold in the Italian market. Dig. Liver Dis..

